# Molecular docking and biological evaluation of some thioxoquinazolin-4(*3H*)-one derivatives as anticancer, antioxidant and anticonvulsant agents

**DOI:** 10.1186/s13065-017-0272-6

**Published:** 2017-05-31

**Authors:** Danah S. Al-Shamary, Monirah A. Al-Alshaikh, Nabila Abdelshafy Kheder, Yahia Nasser Mabkhot, Syed Lal Badshah

**Affiliations:** 1 Women Students-Medical Studies & Sciences Sections, Department of Chemistry, College of Science, King Saud University, P.O. Box 22452, Riyadh, 11495 Saudi Arabia; 2 Department of Chemistry, Faculty of Science, Cairo University, Giza, 12613 Egypt; 3 Department of Pharmaceutical Chemistry, Faculty of Pharmacy, King Khalid University, Abha, 61441 Saudi Arabia; 4 Department of Chemistry, College of Science, King Saud University, P.O. Box 2455, Riyadh, 11451 Saudi Arabia; 5 Department of Chemistry, Islamia College University Peshawar, Peshawar, 25120 Pakistan

**Keywords:** Thioxoquinazolin-4(*3H*)-one, Anticancer activity, Antioxidant activity, Anticonvulsant activity, Molecular docking

## Abstract

**Background:**

The quinazoline are an important class of medicinal compounds that possess a number of biological activities like anticancer, anticonvulsant and antioxidant etc.

**Results:**

We evaluated the previously synthesized quinazoline derivatives **1**–**3** for their anticancer activities against three cancer cell lines (HepG2, MCF-7, and HCT-116). Among the tested compounds, quinazolines **1** and **3** were found to be more potent than the standard drug Vinblastine against HepG2 and MCF-7 cell lines. All the tested compounds had less antioxidant activity and did not exhibit any anticonvulsant activity. Also, molecular docking studies were performed to get an insight into the binding modes of the compounds with human cyclin-dependent kinase 2, butyrylcholinesterase enzyme, human gamma-aminobutyric acid receptor. These compounds showed better docking properties with the CDK2 as compared to the other two enzymes.

**Conclusions:**

The overall study showed that thioxoquinazolines are suitable antitumor agents and they should be explored for other biological activities. Modification in the available lot of quinazoline and synthesis of its novel derivatives is essential to explore the potential of this class of compounds. The increase in the threat and with the emergence of drug resistance, it is important to explore and develop more efficacious drugs.

## Background

**Fig. 1:**
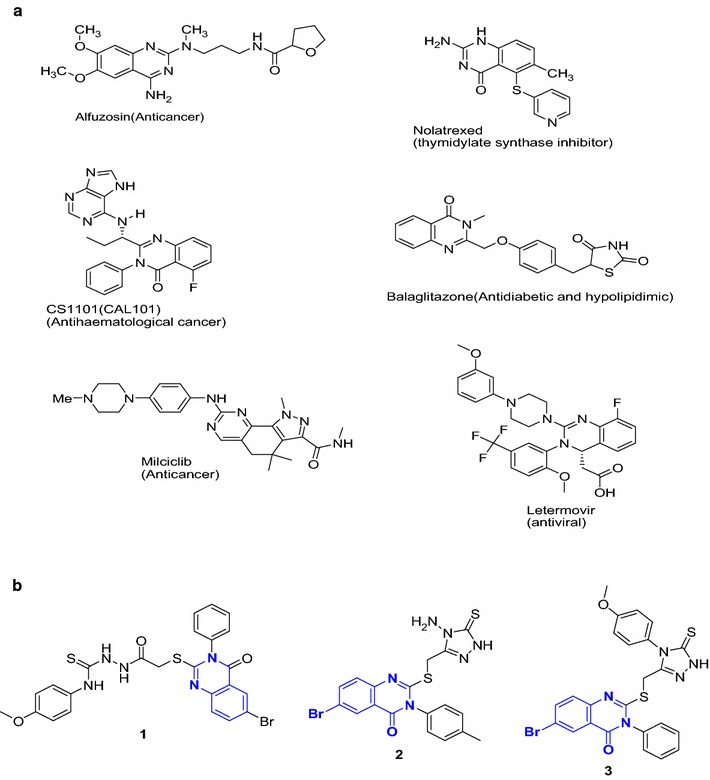
**a** Examples of some the marketed drugs that contain quinazoline ring and their uses. **b** The tested quinazoline derivatives **1**–**3**

The quinazoline moiety containing compounds is of considerable medicinal importance because of their diverse biological activities. It has been observed that they possess anticancer [1–5], antibacterial [6, 7], antifungal [7, 8], antitubercular [9, 10], antiviral [11, 12], anticoccidial [13, 14], anti-inflammatory and analgesics [15–21], antidepressant [22–24], anticonvulsant [23, 24], antimalarial [25, 26], antioxidant [27], antileishmanial [28], neuroprotective [29], antiobesity [30], antihypertensive [31], anti-H_1_-antihistaminic [32], and antiprotozoal activities [33]. The quinazoline moiety is a core unit in a variety of drugs such as Alfuzosin, Nolatrexed, CS 1101 (CAL 101), Balaglitazone, Milciclib, and Letermovir (Fig. 1a). The anticancer activities of quinazolines against different cancer cell lines were reported by different research groups [34–36]. The quinazoline derivatives are potent epidermal growth factor receptor (EGFR) pathway and EGFR tyrosine kinase inhibitors [37–39]. Cancer is one of the devastating and most common life-threatening disease representing a major health problem in both developed and developing countries for the past several decades. The clinical application of chemotherapy for cancer treatment is one of the useful methods, however it has its own limitation due to the severity of the side effects and the development of tumor cell resistance against these cytotoxic agents. Mostly the clinical administration of high doses of anticancer drugs to overcome resistance leads to severe toxicities [40]. Therefore, novel anticancer agents with high potency and reduced toxicity are urgently required to control the plight of cancer and to overcome the drug resistance.

It is reported that during metabolism and respiration in human body, the free radicals and reactive oxygen species (ROS) are produced that causes a number of devastating effects on human health [41, 42]. Over production of ROS is responsible for oxidative damage to DNA that leads to different kinds of cancers [43, 44]. The oxidative damage by free radicals and ROS is blocked by the antioxidants [45]. Antioxidants act by several ways, scavenging free radicals is one of them. To reduce the effects of oxidation on human body, novel and effective antioxidants are required [42]. Here we intended to study the bioactivities of some thioxoquinazolinone derivatives as anticancer, antioxidant and anticonvulsant agents with an aim to find new drugs of synthetic origin. A docking study was performed to fit the proposed quinazolines **1**–**3** into the active site of human cyclin-dependent kinase 2 enzyme, human butyrylcholinesterase enzyme, and human gamma-aminobutyric acid receptor in order to study the interaction between binding model and their anticancer, antioxidant and anticonvulsant activities.

## Methods

### Chemistry

Quinazolinone derivatives were prepared according to the following literature procedures [31, 32].

### Pharmacology

#### Anticancer activity

The compounds were tested for any cytotoxic activity against three tumor cell lines, i.e., liver carcinoma (HepG2), colon cancer (HCT-116) and breast carcinoma (MCF-7) cell lines. When the cells reached confluence (usually 24 h), the cell suspension of the three tumor cell lines were prepared in complete growth medium (DMEM) supplemented with 50 µg/ml gentamycin [33]. The aliquots of 100 μl of cell suspension (1 × 105 cells/ml) were added to each well in a 96-well tissue culture plate. The blank wells contained complete medium in place of cell suspension. The cells were incubated for 24 h at 37 °C in a humidified incubator with 5% CO_2_. After the formation of a complete monolayer cell sheet in each well of the plate, serial twofold dilutions of the tested compounds were added into a 96-tissue culture plate using a multichannel pipette (Eppendorf, Germany). The treated and untreated cells were allowed to grow in the presence of test compounds by further incubating the plates for 24 h. The plates were covered with a plate sealer then incubated at 37 °C. To obtain quantitative cytotoxicity data, the cells were stained with a 0.1% crystal violet solution, then the dye was extracted from the cells by adding glacial acetic acid (33%) to each well and mixed the contents of each well before reading the color absorbance on the ELISA reader (TECAN, Inc, USA) at 490 nm. The absorbance is proportional to the number of surviving cells. We performed each experiment in quadruplicate and repeated three times. The cell growth inhibition (CGI) ratio was calculated from the absorbance values through the following formula:

$$ {\text{CGI }} = \left( {{\text{C}} - {\text{T}}/{\text{C}}} \right) \times 100 $$

where C is mean absorbance value of untreated (control) cells and T is mean absorbance value of treated cells [40, 41].

#### Antioxidant assay

The antioxidant activity of the compounds was determined by the 2,2-diphenyl-1-picrylhydrazyl (DPPH) free radical scavenging assay [48]. Fresh 0.004% (w/v) methanol solution of DPPH was prepared and stored at 10 °C in the dark. A methanol solution of the test compounds were also made. A 40 μl aliquot of the methanol solution of the test compound was added to 3 ml of DPPH solution. Absorbance measurements were recorded immediately with a Milton Roy Spectronic 201 UV–visible spectrophotometer. The decrease in absorbance at 515 nm was determined continuously, with data being recorded at 1 min intervals until the absorbance stabilized (16 min). Ascorbic acid was used as a reference standard and dissolved in distilled water to make the stock solution with the same concentration. The absorbance of the DPPH radical without antioxidant was also measured as control and 95% methanol was used as blank. All the determinations were performed in three replicates and averaged.

% Scavenging of the DPPH free radical was measured using the following equation:

$$ \begin{aligned} \% & {\text{ DPPH radical-scavenging}} \\ & \quad = \frac{ ( {{\text{Absorbance of control}} - {\text{Absorbance of test sample}}} )} {( {\text{Absorbance of control}} )} \times 100. \end{aligned} $$

#### Anticonvulsant activity

The anticonvulsant activity was measured according to the reported methods [42, 43]. A total number of animals used for the study consisted of 53 Wister Albino Mice, 20 adult Wister Albino Rats, and 20 day-old Chicks. Stimulator, constant current unit, and corneal electrode were used for the evaluation of the anticonvulsant activity. All of the under investigation quinazolines compounds were suspended in 30% aqueous solution of PEG 400 and administered intraperitoneally in a volume of 0.01 mg/kg at body weight to the mice. Control animals received 30% aqueous form of PEG 400. The quinazolines **1**−**3** were tested for their anticonvulsant activity against MES-induced seizures and the rotorod toxicity test. Rotorod toxicity test was performed on a 1-in. diameter knurled wooden rod; rotating at 6 rpm.

##### Anticonvulsant effects in the maximal electroshock seizure (MES) test

Maximal electroshock seizures are elicited in mice with a 60-cycle alternating current of 50 mA intensity delivered for 0.2 s via corneal electrodes. A drop of 0.9% saline is introduced in the eye prior to application of the electrodes in order to prevent the death of the animal. Abolition of the hind limb tonic extension component of the seizure indicated protection against the spread of MES-induced seizures.

##### Statistical analysis

The data were expressed as mean ± S.D. The statistical significance of the difference between mean values was determined by Student’s unpaired *t* test. Data were considered statistically significant at a significance level of P < 0.05. The stata statistical analysis package was used for calculation of IC_50_ from the dose response curve.

### Molecular docking

Docking studies were performed using the MOE 2014.09 software package. The protein data bank (PDB) files of the crystal structures of human cyclin-dependent kinase 2 having PDB entry number 1PXO [46], butyrylcholinesterase with PDB ID 4XII and human gamma-aminobutyric acid receptor having PDB ID 4COF were downloaded from the protein data bank website. Regularization and optimization for protein and ligand were performed. Determination of the essential amino acids in binding site were carried out and compared with the present literature. The performance of the docking method was evaluated by redocking the crystal ligands into the assigned active site of the respective enzymes to determine the root mean square deviation (RMSD) values. The interactive docking method was carried out for all the conformers of each compound in the selected active site. Each docked compound was assigned a score according to its fit in the ligand binding pocket (LBP) and its binding mode.

## Results

### Chemistry

Quinazoline derivatives **1**–**3** (Fig. 1b) were synthesized according to the procedures reported previously by our group [31, 32].

### Pharmacology

#### Anticancer activity

**Fig. 2:**
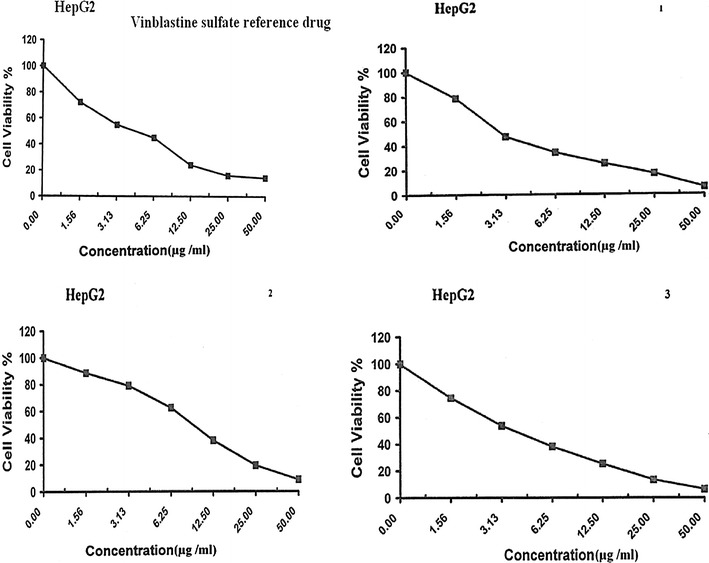
Viability values of quinazoline derivatives **1**–**3** and Vinblastine sulfate against HepG2 cell line

**Fig. 3:**
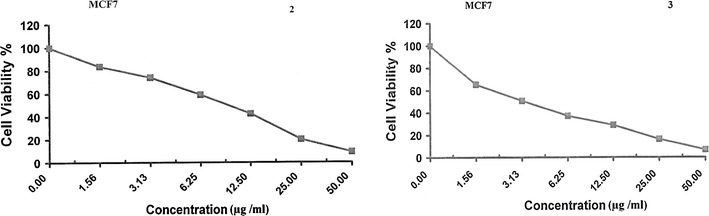
Viability values of quinazoline derivatives **1**–**3** and Vinblastine sulfate against MCF 7 cell line

**Fig. 4:**
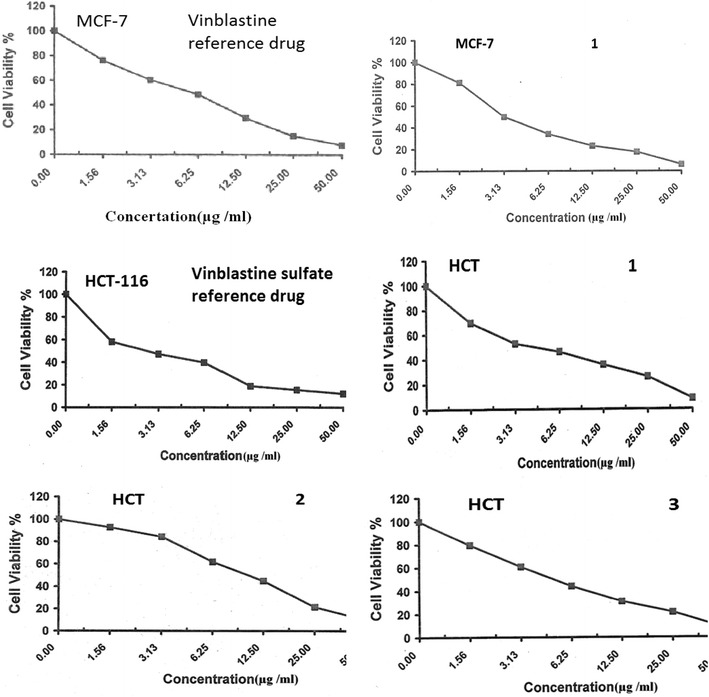
Viability values of quinazoline derivatives **1**–**3** and Vinblastine sulfate against HCT-116 cell line

The liver cancer is ranked in the top ten human cancers worldwide and among the top five of cancers in terms of mortality [44, 45, 47], these information’s motivated us to study the anti-cancer activity of the quinazoline derivatives **1**–**3** against liver carcinoma cell line (HepG2), in addition to colon adenocarcinoma cell lines (HCT-116) and breast carcinoma cell line (MCF-7) using Doxorubicin and Vinblastine sulfate as the positive control drugs [33, 40, 41]. The data generated were used to plot a dose-response curve of which the concentration of test compounds required to kill 50% of the cell population (IC_50_) was determined. The viability values and IC_50_ of quinazolines **1**–**3** against the three tested cell lines are presented in Figs. 2, 3, 4 and Table 1, respectively.

**Table 1:** The inhibitory activities of the tested compounds against three tumor cell lines compared with reference standards

Sample number	IC_50_ (µg/ml)		
	HepG2	MCF-7	HCT-116
1	3.0 ± 0.4	3.1 ± 0.6	4.4 ± 0.9
2	9.5 ± 1.2	9.7 ± 1.8	10.6 ± 2.1
3	3.9 ± 0.6	3.3 ± 0.6	5.7 ± 0.5
Vinblastine sulfate	4.3 ± 0.7	4.6 ± 0.8	2.4 ± 0.3
Doxorubicin	0.5 ± 0.1	0.4 ± 0.1	0.4 ± 0.1

The data are expressed as IC_50_ value ± standard error

The results from Figs. 2, 3, 4 and Table 1 revealed that quinazolines **1** and **3** were more potent than standard drug Vinblastine sulfate against HepG2 and MCF-7 cell lines with IC_50_ values = 3.0, 3.1, and 3.9, 3.3, respectively. However, all the tested compounds were less potent than doxorubicin

#### Antioxidant activity

In the present study, the antioxidant activities of quinazoline derivatives **1**–**3** were tested in vitro by using DPPH radical scavenging percentage compared with ascorbic acid as a reference standard [48] and the results are represented in Table 2. A perusal of the results in Table 2 revealed that all the tested compounds had higher IC_50_ value compared with the reference standard ascorbic acid.

**Table 2:** The in vitro antioxidant activity of quinazolines **1**–**3** in DPPH method

Sample number	IC_50_
1	78 ± 4
2	312 ± 13
3	124 ± 9
Ascorbic acid	11 ± 2

The data are expressed as IC_50_ value (µg/ml) ± standard error

#### Anticonvulsion activity

Convulsion was induced in different animal models using maximum electric shock test [42, 43]. Unfortunately, the three compounds showed no anticonvulsant activity when its potency was compared with that of the reference drug, phenytoin (Table 3).

**Table 3:** Quantitative anticonvulsant data for mice using maximal electroshock test

Sample number	Maximal electroshock ED50 (mg/kg)
1	>200
2	>200
3	>200
Phenytoin standard	10.3 ± 0.6

### Molecular docking

All dock runs were conducted using MOE 2014.09 software.

#### The binding mode of the quinazoline derivatives 1–3 with the human cyclin-dependent kinase 2

**Fig. 5:**
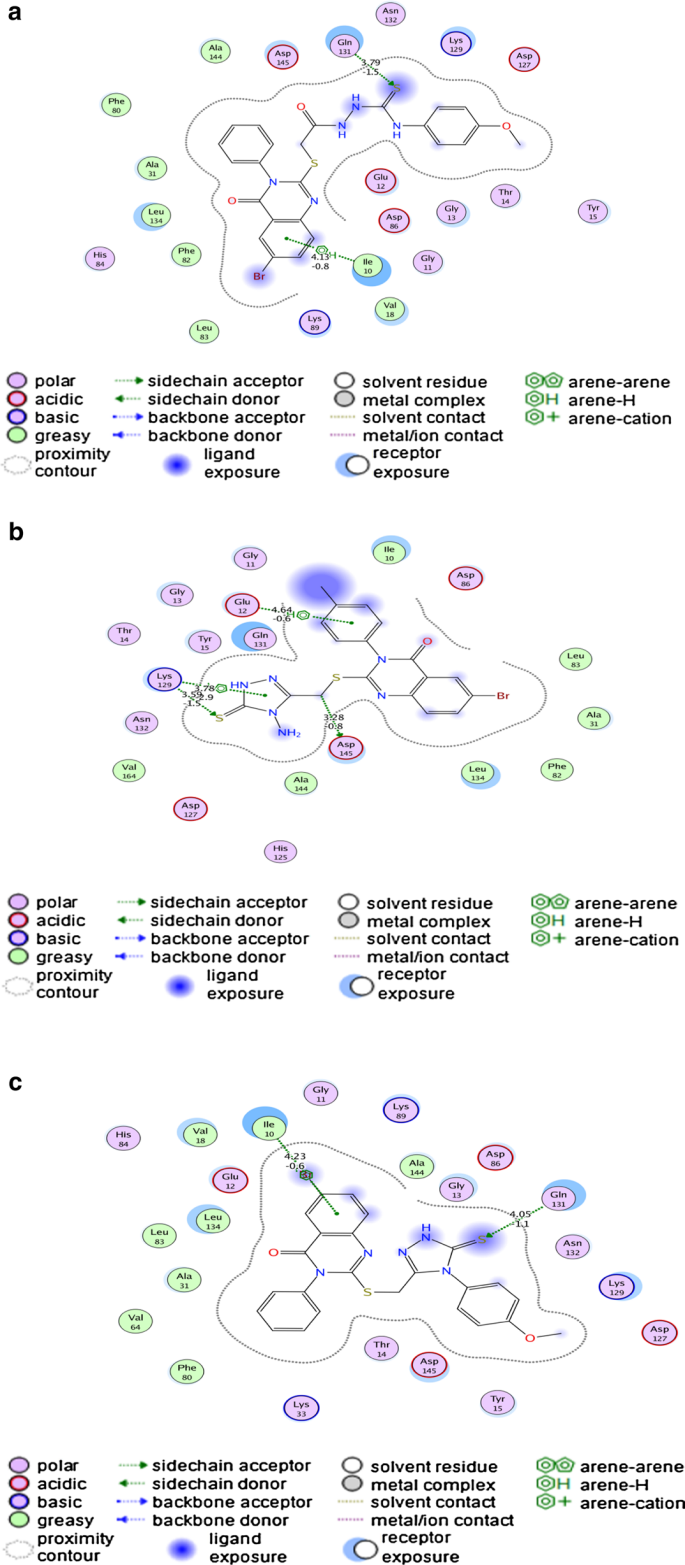
**a** 2-D representation of docking of quinazoline **1** into human cyclin-dependent kinase 2 enzyme. **b** 2-D representation of docking of quinazoline **2** into human cyclin-dependent kinase 2 enzyme. **c** 2-D representation of docking of quinazoline 3 into human cyclin dependent kinase 2 enzyme

The docking of the quinazolines **1**–**3** into the active site of human cyclin-dependent kinase 2 enzyme were conducted to get information about the interaction of these compounds inside the kinase. The docking results of quinazoline **1** into the active site of human cyclin-dependent kinase 2 enzyme showed arene-hydrogen interaction with bond length of 4.13 Å and binding energy of −0.8 (kcal/mol) with Ile10, and hydrogen bond between thiocarbonyl of the ligand as a hydrogen bond acceptor and Gln131 with bond length of 3.79 Å and binding energy of −1.5 (kcal/mol) (Fig. 5a). These interactions were quite favorable due to negative free energy and suitable bond lengths.

The molecular docking study of quinazoline **2** into the binding pocket of human cyclin-dependent kinase 2 enzyme revealed two interactions; arene-cation interaction with bond length of 3.78 Å and binding energy of −2.9 (kcal/mol) and hydrogen bond acceptor interaction with bond length of 3.59 Å and binding energy of −1.5 (kcal/mol) with Lys129. It also showed a hydrogen donor interaction with bond length of 3.28 Å and binding energy of −0.8 (kcal/mol) with Asp145, in addition to arene-hydrogen interaction with bond length of 4.64 Å and binding energy of −0.6 (kcal/Mol) with Glu12 (Fig. 5b).

Alignment study of docked quinazoline **3** into the active binding pocket of the human cyclin-dependent kinase 2 enzyme (Fig. 5c) revealed arene-hydrogen interaction with bond length of 4.23 Å and binding energy of −0.6 (kcal/mol) with Ile10. There was a hydrogen acceptor interaction between Gln131 and one of the sulphur atom of the compound with bond length of 4.05 Å and binding energy of −1.1 kcal/mol.

#### The binding mode of the quinazoline derivatives 1−3 with the human butyrylcholinesterase

**Fig. 6:**
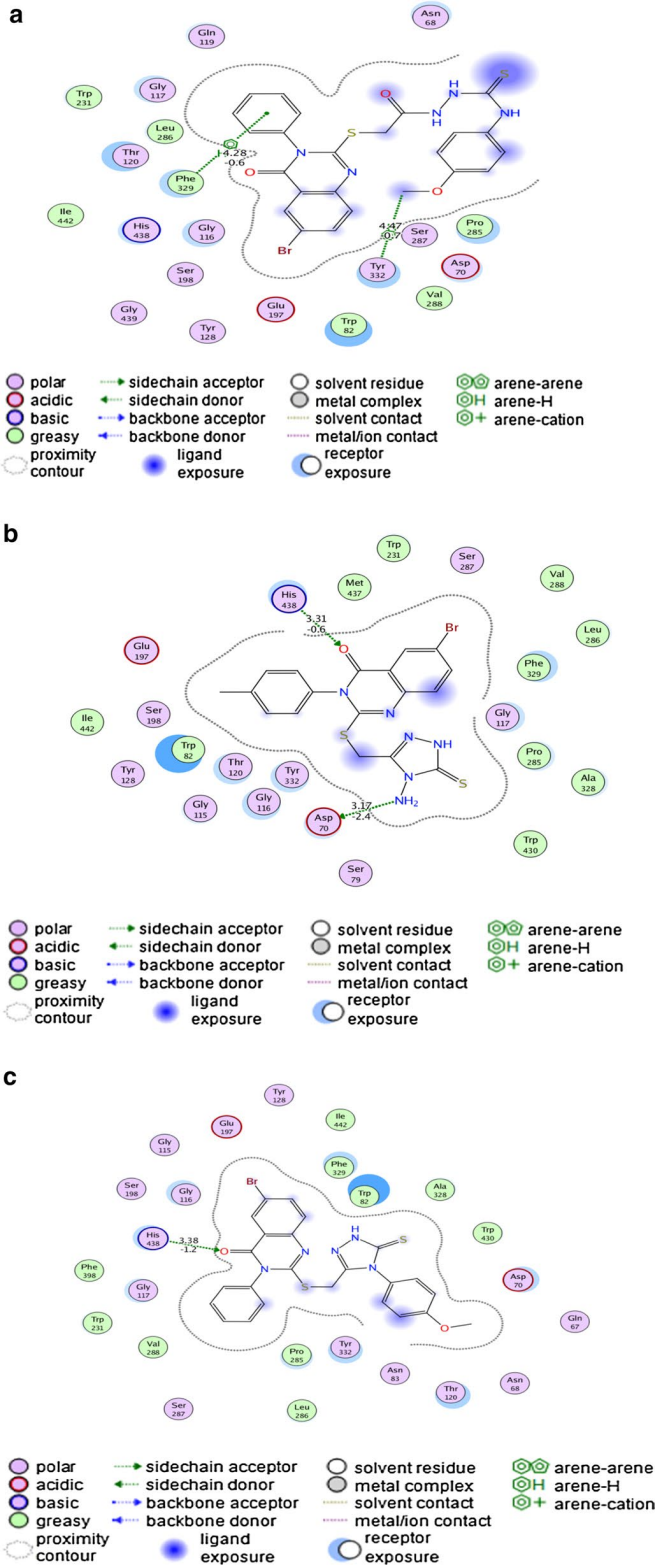
**a** 2-D representation of docking of quinazoline **1** into butyrylcholinesterase. **b** 2-D representation of docking of quinazoline **2** into butyrylcholinesterase. **c** 2-D representation of docking of quinazoline **3** with butyrylcholinesterase

The docking results of quinazoline **1** with the human butyrylcholinesterase showed arene–arene interaction between the side benzene ring of compound **1** and Phe329 with bond length of 4.28 Å and binding energy of −0.6 kcal/mol. The second interaction is that of a hydrogen-arene interaction between hydroxyl group of the compound and Tyr332 with bond length of 4.47 Å and binding energy −0.7 (kcal/mol) for this interaction (Fig. 6a). The molecular docking studies of the quinazoline **2** into the human butyrylcholinesterase showed hydrogen donor interaction between amine group and Asp70 having bond length of 3.17 Å and binding energy of −2.4 kcal/mol. There is also a hydrogen acceptor interaction between His438 and keto group of quinazoline **2** resulting in a bond length of 3.31 Å and binding energy of −0.6 kcal/mol as shown in Fig. 6b. In a similar manner, an alignment study of docked quinazoline **3** into the active binding pocket of butyrylcholinesterase revealed a hydrogen acceptor interaction with bond length of 3.38 Å and binding energy of −1.2 (kcal/mol) between the keto group and His438 (Fig. 6c). These docking studies showed strong interactions between the quinazoline analogues and the butyrylcholinesterase and they may have physiological significance.

#### The binding mode of the quinazoline derivatives 1–3 with human gamma-aminobutyric acid receptor

**Fig. 7:**
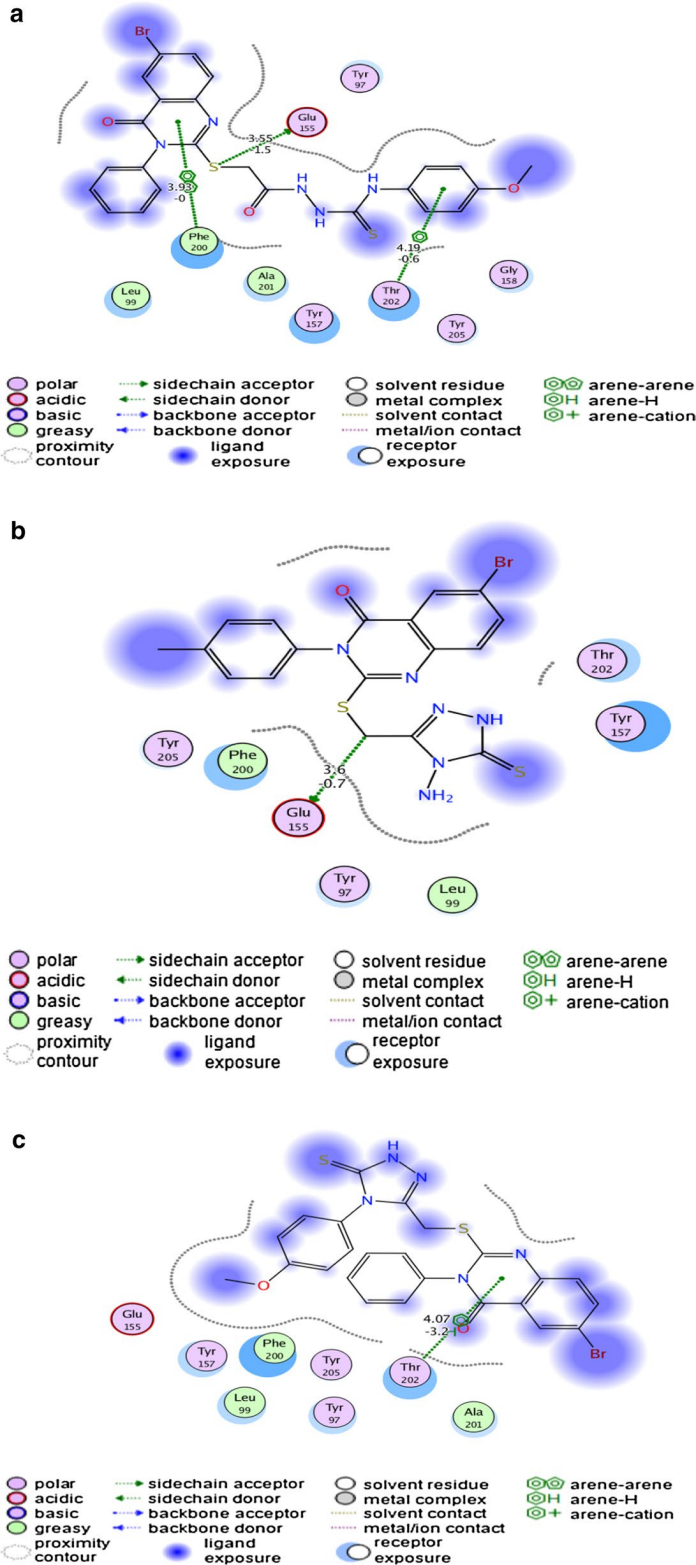
**a** 2-D representation of docking of quinazoline **1** into the human gamma-aminobutyric acid receptor. **b** 2-D representation showing interactions between human gamma-aminobutyric acid receptor and the quinazoline **2**. **c** 2-D representation showing interactions between human gamma-aminobutyric acid receptor and the compound **3**

The docking results of the quinazoline **1** with the human gamma-aminobutyric acid receptor showed arene-hydrogen interaction with bond length of 4.19 Å and binding energy of −0.6 (kcal/mol) with Thr202 of the receptor protein. The arene–arene interaction was established between Phe200 and the pyrimidine ring of the ligand with bond length of 3.93 Å and has a binding energy of −0.0 kcal/mol. The third type of interaction is side chain donor between Glu155 and the bridging sulphur atom of the ligand having bond length of 3.55 Å and binding energy of −1.5 kcal/mol (Fig. 7a). Thus the compound **1** showed favorable interactions inside the active pocket. In a similar manner docking of quinazoline **2** showed hydrogen donor interaction with bond length of 3.60 Å and binding energy of −0.7 kcal/mol with Glu155 (Fig. 7b). The docking study of the docked compound **3** into the active binding pocket of the human gamma-aminobutyric acid receptor showed arene-hydrogen interaction with bond length of 4.07 Å with binding energy of −3.2 kcal/mol with Thr202 of the receptor (Fig. 7c). Thus all the three analogues of quinazolines makes favorable interactions inside the active site of the human gamma-aminobutyric acid receptor and they are possible ligands of it.

### Drug-likeness analysis

The drug-like properties were calculated and the results were summarized in Table 4. The drug-like properties consist of molecular weight (MW), octanol–water partitioning coefficient (AlogP) based on Ghose and Crippen’s methods [49, 50]. The number of hydrogen bond acceptors (HBA), the number of hydrogen bond donors (HBD) and total polar surface area (TPSA). All the data were calculated using the MOE 2014.09 package. Results of Table 4 revealed that quinazoline **2** obeyed the Lipinski rule of five in drug-likeness test [51].

**Table 4:** Drug-like properties of the quinazolines **1**–**3**

Sample number	Molecular weight (g/mol)	TPSA	LogS	LogP	HBA	HBD
1	570.49	127.15	−9.26	4.86	5	3
2	475.39	118.41	−8.31	3.52	5	2
3	552.48	101.62	−9.63	5.55	5	1

## Discussion

We tested the three thioxoquinazolines derivative compounds on three different types of cancer cells and they all showed cytotoxicity to them. These thioxoquinazolines were active against the cancer cell lines in different concentrations. The molecular docking studies of the thioxoquinazoline derivatives with the human cyclin dependent kinase showed several interactions and have favorable docking free energies. These docking studies of quinazoline with cyclin dependent kinase 2 are in agreement with other studies [52–54]. Further these analogues also showed favorable interactions inside the active site of human butyrylcholinesterase and gamma-aminobutyric acid receptor. The quinazolines analogues are also working as an antioxidants and they showed IC_50_ values between 78 μg/ml and 312 μg/ml as compared to the standard ascorbic acid that has a IC_50_ of 11 μg/ml. Although they are not as much potent antioxidant as ascorbic acid but their antioxidant properties can be increased by attaching suitable substituents with the quinazoline nucleus [55, 56]. Some quinazolines also posses anticonvulsant activities [57] and that is why we tested our synthesized compound for this purpose but unfortunately we did not observe such properties. Therefore, it is necessary to screen such quinazoline compounds for a number of biological activities.

## Conclusions

The results showed that the quinazolinones **1** and **3** were more potent than standard drug Vinblastine sulfate against HepG2 and MCF-7 cell lines, all the tested compounds had low antioxidant activity compared with the reference standard ascorbic acid. In the near future, it will be better to utilized QSAR and virtual screening methods to design and select more suitable quinazoline ligands that posses better anticancer and antioxidant activities. The three tested compounds here showed no anticonvulsant activity. This work on testing thioxoquinazoline for biological activities is an initial effort and these and other synthesized compounds will be tested for antimicrobial, antiviral and antimalarial activities.
